# Integrated assays with *in silico* analyses indicated the biocontrol potential of *Bacillus velezensis* BJ1 against fungal phytopathogens

**DOI:** 10.1128/spectrum.00944-26

**Published:** 2026-10-14

**Authors:** Yonghua Li, Manawaduge Ayesha Madushani, Mei Liu, Chenfang Wang, Hui Wang, Xinghong Li, Kevin David Hyde, Wei Zhang, Jiye Yan

**Affiliations:** 1 Beijing Key Laboratory of Environment Friendly Management on Fruit Diseases and Pests in North China; Key Laboratory of Environment Friendly Management on Fruit and Vegetable Pests in North China (Co-construction by Ministry and Province), Ministry of Agriculture and Rural Affairs, Institute of Plant Protection, Beijing Academy of Agriculture and Forestry Sciences 107624 , Beijing, China; 2 Center of Excellence in Fungal Research, Mae Fah Luang University 68004, Chiang Rai, Thailand; 3 School of Science, Mae Fah Luang University 295535, Chiang Rai, Thailand; Agroscope, Nyon, Switzerland

**Keywords:** biocontrol, *Botrytis cinerea*, genome mining, gray mold, plant disease management, sustainable agriculture

## Abstract

Fungal diseases continue to devastate global fruit and vegetable production, emphasizing the urgent need for sustainable disease management approaches such as microbial biocontrol. In this study, a locally isolated bacterial strain was identified as *Bacillus velezensis* BJ1 based on phylogenomic analysis, and its biocontrol potential was evaluated using *in vitro*, *in vivo*, and *in planta* assays combined with *in silico* analysis. *In vitro* dual culture assay demonstrated broad-spectrum antagonistic activity of the BJ1 strain against 59 isolates of common fungal phytopathogens affecting fruit trees and vegetables, with inhibition rates ranging from 53.76% to 98.45%. The strongest suppression was observed against *Diaporthe sojae* OSS① (98.45%), followed by *D. sojae* B2 JZB320190 (89.15%), *Cladosporium cladosporioides* TZZG-11-A-S1 (85.37%), *C. cladosporioides* TZZG-6-A-S1 (85.22%), *D. sojae* JZB320189 (80.62%), and *D. eres* TZZG36-S1 (80.62%). Detached tomato leaf assays against the gray mold pathogen *Botrytis cinerea* demonstrated that BJ1 achieved high control efficiencies of 87.87% and 68.95% for therapeutic and preventive treatments, respectively, at a concentration of 1 × 10⁷ CFU/mL. Furthermore, a BJ1-based water-dispersible granule formulation applied at 2 × 10^8^ CFU/mL achieved an 80.48% disease inhibition rate under greenhouse conditions. Genome mining identified 13 biosynthetic gene clusters encoding antimicrobial compounds, suggesting a genetic basis for BJ1’s biocontrol activity. Overall, *B. velezensis* BJ1 demonstrates strong potential for development as an effective biocontrol product, offering both genomic insights and practical application in sustainable disease management.

**IMPORTANCE:**

Biological control is a sustainable strategy for managing plant pathogens while reducing the environmental and health risks associated with chemical pesticides. However, their practical application is often limited by poor environmental adaptation, limited broad-spectrum activity, and inconsistent efficacy. *Bacillus velezensis* BJ1, a locally isolated strain, exhibited broad antagonistic activity against 59 isolates of common phytopathogens across eight host plants. Further, it exhibited consistent *in vivo* and *in planta* efficacy against the highly virulent pathogen *Botrytis cinerea* under both protective and therapeutic treatments, highlighting its potential for both disease prevention and post-infection suppression. Whole-genome phylogenomic analysis confirmed its precise taxonomic identity, and genome mining suggested multiple biosynthetic gene clusters potentially associated with antifungal activity and plant protection. This study highlights BJ1 as a robust and eco-friendly candidate for sustainable disease management.

## INTRODUCTION

Plant diseases threaten the fundamental human right to sufficient and safe food, leaving around 800 million people worldwide suffering from hunger or malnutrition (1, 2). These diseases cause 13%–22% of annual crop yield losses and impose high costs on food production and disease management (2). Over the past decades, disease management has relied mainly on synthetic chemical pesticides; however, their widespread use is associated with environmental contamination, development of pesticide resistance, and potential health risks to humans and animals (3). Therefore, there is a growing need for research into effective and eco-friendly alternatives to safeguard crops from plant diseases, minimizing intensive use of agrochemicals.

Biological control offers great potential for sustainable disease management, utilizing living organisms or their derivatives (4). Among the microbial antagonists, *Bacillus* species have been extensively studied due to their effective ability in plant growth promotion, plant protection against a broad spectrum of phytopathogens, and the possibility of developing stable commercial products (5–7). These agents suppress pathogens through both direct and indirect mechanisms. Direct mechanisms include competition for nutrients and space, antibiosis via the production of toxic secondary metabolites such as lipopeptides, bacteriocins, antibiotics, biosurfactants, and volatile compounds, as well as the secretion of lytic enzymes that inhibit spore germination and degrade germ tubes. Indirect mechanisms enhance plant resilience by inducing systemic resistance, promoting plant growth and nutrient uptake, and improving soil fertility and stress tolerance (8–11).

Several *Bacillus*-based biocontrol agents, including *B. velezensis*, *B. subtilis*, *B. amyloliquefaciens*, *B. halotolerans*, and *B. thuringiensis*, were reported to remarkably control plant pathogens by secreting a variety of antimicrobial compounds, including antimicrobial lipopeptides (LPs), polyketides (PKs), volatiles, and lytic enzymes through colonization (11–14), and even very low concentrations of these antibiotic substances or other metabolites could inhibit the plant pathogens (11, 15). These metabolites are produced through specialized biosynthetic pathways encoded by physically clustered sets of genes in the genome, referred to as biosynthetic gene clusters (BGCs) (16). Different structural classes of BGCs exist, including non-ribosomal peptide synthetases (NRPS), polyketide synthases (PKS), terpenes, and bacteriocins (17). NRPS and PKS are popular targets for natural product discovery as they are known to synthesize a diversity of antimicrobials with the potential of producing enormous biocontrol derivatives in microbes (18). The advent of DNA sequencing technologies and computational genome mining tools facilitates complete genome sequencing of potential biocontrol strains and thereby identifies their biosynthetic gene clusters encoding novel antimicrobial compounds (17, 19).

*Bacillus velezensis* strains were reported to remarkably control economically important crop diseases, including powdery mildew (20), wheat head blight (21, 22), *Fusarium* wilt (23), potato late blight (24, 25), potato scab (26), and cherry shot hole disease (27). Beyond its biocontrol ability, *B. velezensis* strains are documented for their roles in promoting plant growth, abiotic stress tolerance, and soil bioremediation (28–31).

Although fruit and vegetable crops are affected by a wide array of fungal pathogens, most existing biocontrol studies focus on a narrow range of pathogens, rely largely on laboratory-based evaluations, and show inconsistent field performance. This emphasizes the need for a comprehensive assessment of biocontrol agents under diverse experimental conditions to evaluate their reliability and broader applicability in agricultural disease management. In this study, we report the comprehensive characterization of a locally isolated bacterial strain, *B. velezensis* BJ1, with precise taxonomic identification through phylogenomic analysis, and evaluate its potential as a broad-spectrum biocontrol agent using an integrative experimental approach combining *in vitro*, *in vivo*, and *in planta* assays together with genome-mining analyses. Overall, this work provides insights into the biocontrol potential of *B. velezensis* BJ1 and supports its prospective application in managing fungal diseases in agricultural systems.

## MATERIALS AND METHODS

### Isolation and morphological characterization of BJ1

Our study was initially designed to identify pathogens associated with grapevine trunk diseases from grapevine tissues collected in Haidian District, Beijing, China. However, during the tissue-based isolation process (32), a bacterial isolate (BJ1) exhibiting strong antagonistic activity against the pathogenic fungi was identified. This bacterium was purified by streaking onto Lysogeny Broth (LB) agar plates and incubated at 28°C for 2–3 days to observe the growth and the colony characteristics. The morphology and size of single cells of strain BJ1 were examined using a scanning electron microscope (HITACHI SU8010, Japan), following the method in Madushani et al. (12).

### Genomic DNA extraction, sequencing, assembling, and annotation of BJ1

Genomic DNA of strain BJ1 was extracted using the SDS method (33), detected by agarose gel electrophoresis, and quantified using a Qubit 2.0 Fluorometer (Thermo Scientific). Whole-genome sequencing was conducted using a hybrid PacBio Sequel and Illumina NovaSeq PE150 strategy (performed by Beijing Novogene Bioinformatics Technology Co., Ltd.) to leverage the PacBio long read advantage of contiguous assembly and Illumina’s higher per-base accuracy for polishing. Genome assembly, polishing, and quality assessment of strain BJ1 were performed following the same procedure described in Madushani et al. (12). Briefly, genome assembly was performed using SMRT Link v5.0.1, with PacBio reads shorter than 500 bp removed prior to assembly. The preliminary assembly was polished using the Arrow algorithm, followed by secondary error correction with Illumina reads aligned using BWA (34). Assembly completeness was evaluated using BUSCO based on the *firmicutes_odb10* database, and GC content and sequencing depth were assessed to examine genome quality and potential contamination (35). The unimodal patterns observed in GC content and read abundance suggested that the data were free from contamination. The absence of plasmid sequences was assessed by comparing the genome sequence against plasmid sequences in the NCBI database using BLASTn.

Protein-coding genes were predicted and curated using GeneMarkS v4.17 (36). Functional annotation of the predicted open reading frames (ORFs) was carried out by alignment against several databases, including Gene Ontology (GO) (http://geneontology.org/), Cluster of Orthologous Groups (COG/KOG) (https://www.ncbi.nlm.nih.gov/research/COG), eggNOG (evolutionary genealogy of genes: Non-supervised Orthologous Groups) (http://eggnog6.embl.de/), Kyoto Encyclopedia of Genes and Genomes (KEGG) (http://www.genome.jp/kegg/), the non-redundant (NR) protein database (https://www.ncbi.nlm.nih.gov/protein), Swiss-Prot (https://www.uniprot.org/), the carbohydrate-active enzymes (CAZy) (http://www.cazy.org/), and Transporter Classification Database (TCDB) (http://www.tcdb.org).

### Phylogenomic-based taxonomic identification of BJ1

The taxonomic identity of BJ1 was determined by the Type (strain) Genome Server (TYGS) platform (https://tygs.dsmz.de), based on the whole-genome sequence of the BJ1 strain and comparisons with *Bacillus* type strains (37). The digital DNA–DNA hybridization (dDDH) values from pairwise comparisons between strain BJ1 and reference genomes were obtained directly from the TYGS analysis results (37, 38). In addition, *Bacillus* type and reference genomes were obtained from the NCBI Genome database, and pairwise average nucleotide identity (ANI) values between BJ1 and those reference genomes were calculated using the OrthoANIu algorithm in the EZBioCloud ANI calculator (https://www.ezbiocloud.net/tools/ani) (39). The final taxonomic assignment of the strain BJ1 was determined based on the established ANI and dDDH threshold values.

### *In silico* prediction of the biocontrol potential of BJ1

The genomic potential of strain BJ1 for antimicrobial secondary metabolite biosynthesis was evaluated by identifying biosynthetic gene clusters (BGCs) using the antiSMASH 7.0 online server with “relaxed” parameters and default analysis modules (ClusterBlast and KnownClusterBlast) (17). Biosynthetic gene clusters exhibiting less than 70% amino acid identity with known clusters were identified as novel (19). The functions of the resulting metabolites were inferred based on database annotations and previous literature (12, 17).

### *In vitro* antagonistic activity of BJ1 against phytopathogenic fungi

The antagonistic activity of strain BJ1 was evaluated using the *in vitro* dual culture assay, following the method described by Favre-Godal et al. (40). A total of 59 fungal phytopathogen isolates obtained from the culture collection of the Beijing Academy of Agriculture and Forestry Sciences were used in this study. These pathogens are major causal agents of economically important diseases, such as anthracnose, black foot disease, *Botryosphaeria* dieback, gray mold, root rot, leaf spot, *Fusarium* wilt, and *Sclerotinia* stem rot, isolated from multiple agricultural host plants, including grapevine, cherry, strawberry, watermelon, chili, Chinese chives, eggplant, and tomato. All the fungal pathogens were cultured on PDA plates and incubated at 28°C for 3–5 days. Strain BJ1 was first cultured on LB agar plates at 25°C for 2–3 days. A single colony was then inoculated into LB broth and incubated at 37°C, 200 rpm for 8–10 h. The concentration of the entire fermentation broth was adjusted to OD_600_ = 0.1 and freshly prepared prior to each experiment as the BJ1 bacterial inoculum.

Two mycelial plugs (5 mm in diameter) of the same fungal pathogenic strain and two sterilized filter paper discs (6 mm in diameter) were positioned approximately 1 cm from the edge of a new PDA plate in opposite directions. Each disc was loaded with 4 µL of BJ1 bacterial suspension (OD_600_ = 0.1). In addition, the control plates contained two mycelial plugs of the same fungal pathogen and two sterile filter paper discs loaded with 4 µL of sterile water instead of the BJ1 suspension. Each treatment consisted of three biological replicates, with each replicate represented by an independently prepared Petri plate. The plates were randomly arranged in the incubator and maintained at 28°C for 5 days. The resulting radial colony growth (C) of the fungal pathogen was measured on both the control and BJ1-treated plates to determine the pathogen growth inhibition rate.


$$Inhibition\;rate\;\left(\%\right)=\;\frac{\left(Ccontrol-\;Ctreatment\right)}{\;Ccontrol}\times 100\%$$

### *In vivo* preventive and therapeutic efficacy of BJ1 against *Botrytis cinerea*

After evaluating the antagonistic activity of the *B. velezensis* BJ1 strain, its preventive and therapeutic effects were further assessed against the virulent pathogen *Botrytis cinerea* (causal agent of tomato gray mold disease) using an *in vivo* leaf inoculation assay (15, 41). The treatments comprised four *B. velezensis* BJ1 concentrations, a positive control (*Bacillus subtilis*), and a negative control (sterile distilled water). The entire fermentation broth of BJ1 (OD₆₀₀ = 0.1) was diluted with sterile distilled water to prepare 200 mL of each of four bacterial suspensions at concentrations of 1 × 10⁵, 1 × 10⁶, 5 × 10⁶, and 1 × 10⁷ CFU/mL. *B. subtilis* wettable powder (1 × 10^10^ CFU/g; Jiangxi Zheng Bang Crop Protection Co., Ltd., China; pesticide registration no. PD20151587) was suspended in sterile distilled water to prepare 200 mL of a 1 × 10⁸ CFU/mL suspension. Healthy, similarly sized leaves in the middle and lower positions of fruited tomato plants (Tomato cultivar Yingfen 8) were collected from greenhouses on the day of the inoculation experiment. The collected leaves were surface-cleaned with running water, sterilized in 70% ethanol for 30 s, and rinsed three times with sterile water. The petioles were then wrapped with sterile, moistened cotton, and the leaves were allowed to air-dry under sterile conditions. These leaves were subjected to an antifungal assay by slightly modifying the method of Du et al. (42). Each treatment consisted of four biological replicates, with each replicate comprising six detached leaves placed in a separate crisper box containing double-layered sterile filter paper. Each crisper box was considered an experimental unit. In order to assess the protective effect of the BJ1 strain, leaves were treated with 200 mL of each treatment using a hand-held pressure sprayer (Food and Drug Administration Medical Device 20180014; Aoxiang Medical Equipment Factory, Taihou, Zhejiang, China) until all the leaf surfaces were completely wetted. All treatments were applied under identical spraying conditions to ensure consistent coverage among replicates. Then, the crisper boxes containing treated leaves were placed in an artificial humid chamber and incubated at 25°C and 60% relative humidity. After 24 h of incubation, the leaves were inoculated by placing the mycelial plug (4 mm in diameter) of *B. cinerea* (5 days old) on the leaves (non-wound inoculation method). The crisper boxes were randomly arranged within the artificial humid chamber at 25°C and 60% relative humidity for 3–5 days. Additional controls consisting of leaves treated with the four BJ1 concentrations and inoculated with sterile PDA plugs were included to confirm the absence of phytotoxic effects caused by the BJ1 strain. The therapeutic effect of strain BJ1 was determined by inoculating the leaves with the pathogen first, and then treating them with different concentrations of BJ1, the negative control, and the positive control at 48 h post-inoculation of the pathogen. The methods used were the same as those used to determine the protective effect. The entire experiment was repeated three times. Infection symptoms were observed, and the average lesion diameter of each leaf was measured at 3–5 days after inoculation. The control effect calculation formula is as follows:


$$Control\;effect\;\left(\%\right)\;=\;\frac{\left(Average\;lesion\;diameter\;of\;control\;-Average\;lesion\;diameter\;of\;treatment\right)}{\left(Average\;lesion\;diameter\;of\;control\;-\;Average\;diameter\;of\;mycelial\;plug\right)}\times 100\%$$

### *In planta* therapeutic efficacy of BJ1 against *Botrytis cinerea*

The BJ1 bacterial strain was further evaluated for its disease-controlling ability against *Botrytis cinerea* of tomato gray mold disease using an *in planta* experiment. A greenhouse with fruited tomato cultivation (Tomato cultivar JingCai 8) infested with tomato gray mold disease was selected for the experiment. The treatments comprised three *B. velezensis* BJ1 concentrations, a positive control (*Bacillus subtilis*), and a negative control (water). The entire fermentation broth of BJ1 (OD_600_ = 0.1) was mixed with a carrier, dispersant, wetting agent, and stabilizer, then ground into powder by vacuum spray-drying to obtain water-dispersible granules. Then, the BJ1 water-dispersible granules (Concentration 2 × 10^10^ CFU/g) were dissolved in sterile distilled water and prepared at three concentrations of 5 × 10^7^, 1 × 10^8^, and 2 × 10^8^ CFU/mL. *B. subtilis* wettable powder (1 × 10^10^ CFU/g; Jiangxi Zheng Bang Crop Protection Co., Ltd., China; pesticide registration no. PD20151587) was suspended in sterile distilled water to prepare a 1.56 × 10⁸ CFU/mL suspension. Each treatment comprised four biological replicate plots, with each experimental plot (20 m²) considered as one independent replicate. Treatments were randomly assigned to the plots within the greenhouse. Each treatment was diluted with water to a final spray volume of 675 L ha⁻¹ and sprayed uniformly until complete leaf coverage and slight runoff. The spray was applied three times at the early stage of tomato gray mold using an electric hand-held sprayer (SX-MD16E-2L, Shixia Sprayer Co., Ltd., Taizhou, Zhejiang, China). All treatments were applied under identical spraying conditions to ensure consistent coverage among replicates. No irrigation, fertilization, or additional pesticide application was performed during the experiment. Disease assessments were conducted on plants within each plot. The disease incidence and control effectiveness were assessed using the following formula:


$$\;\;\;\;\;\;\;\;\;Disease\;incidence\;\left(\%\right)\;=\;\frac{Number\;of\;diseased\;plants}{Total\;number\;of\;plants\;investigated}\times 100\%$$

$$Control\;effect\;(\%)=1-\frac{\left(Disease\;incidence\;of\;control\;before\;irrigated\;by\;water\times disease\;incidence\;of\;treatment\;after\;irrigated\;by\;microbial\;pesticide\right)}{\left(Disease\;incidence\;of\;control\;after\;irrigated\;by\;water\times disease\;incidence\;of\;treatment\;before\;irrigated\;by\;microbial\;pesticide\right)}\times 100\%$$

### Data analysis

The same statistical approach was applied to all the experiments (dual culture assay, leaf inoculation assay, and *in planta* assay). All the data were checked for normality and homogeneity of variance using Shapiro–Wilk and Levene’s tests, respectively. As these assumptions were met, no data transformation was required. Data were analyzed using one-way analysis of variance (ANOVA), followed by Tukey’s HSD post-hoc test using SPSS version 18.0 (IBM Corp., Armonk, NY, USA). Differences were considered statistically significant at *P* < 0.05.

## RESULTS

### Morphological characterization of BJ1

**Fig 1:**
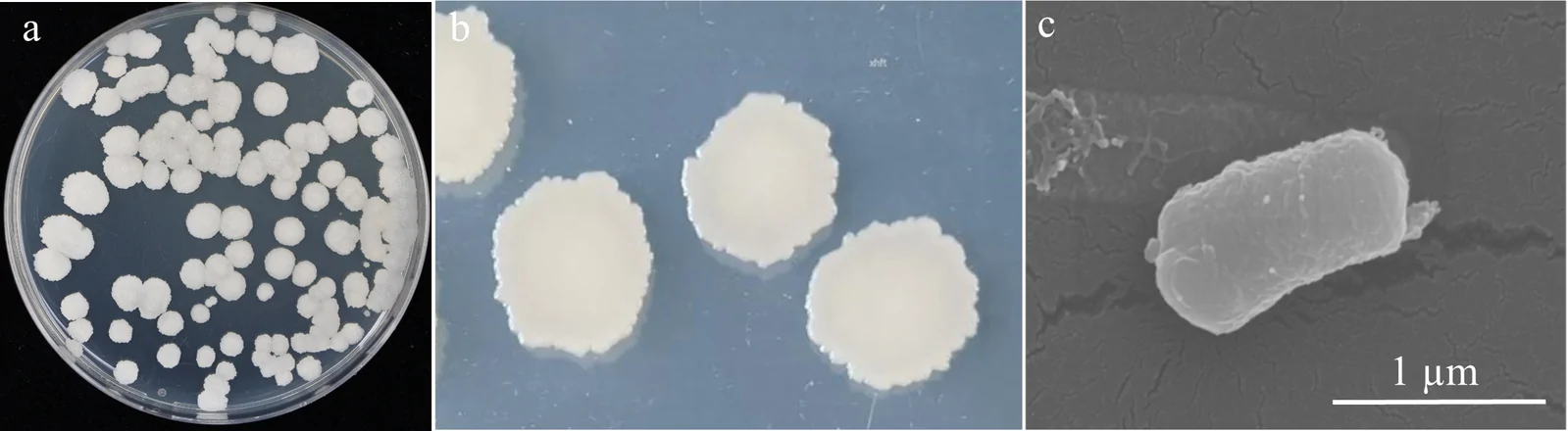
Morphological characteristics of the colony and cells of *Bacillus velezensis* strain BJ1. (**a–b**) Colony morphology of strain BJ1 after 2 days of incubation at 28°C on LB medium. (**c**) Scanning electron micrograph depicting the cellular morphology of strain BJ1 (Scale bar: c = 1 µm; magnification: c = 40,000× ).

The strain BJ1 grew well on LB medium at 28°C after 2 days. BJ1 cells were rod-shaped, and the colonies were circular or nearly circular in shape, with a milky-white appearance. Colony margins were uneven, and the surface exhibited slight wrinkling (Fig. 1).

### Genomic characterization of BJ1

**Fig 2:**
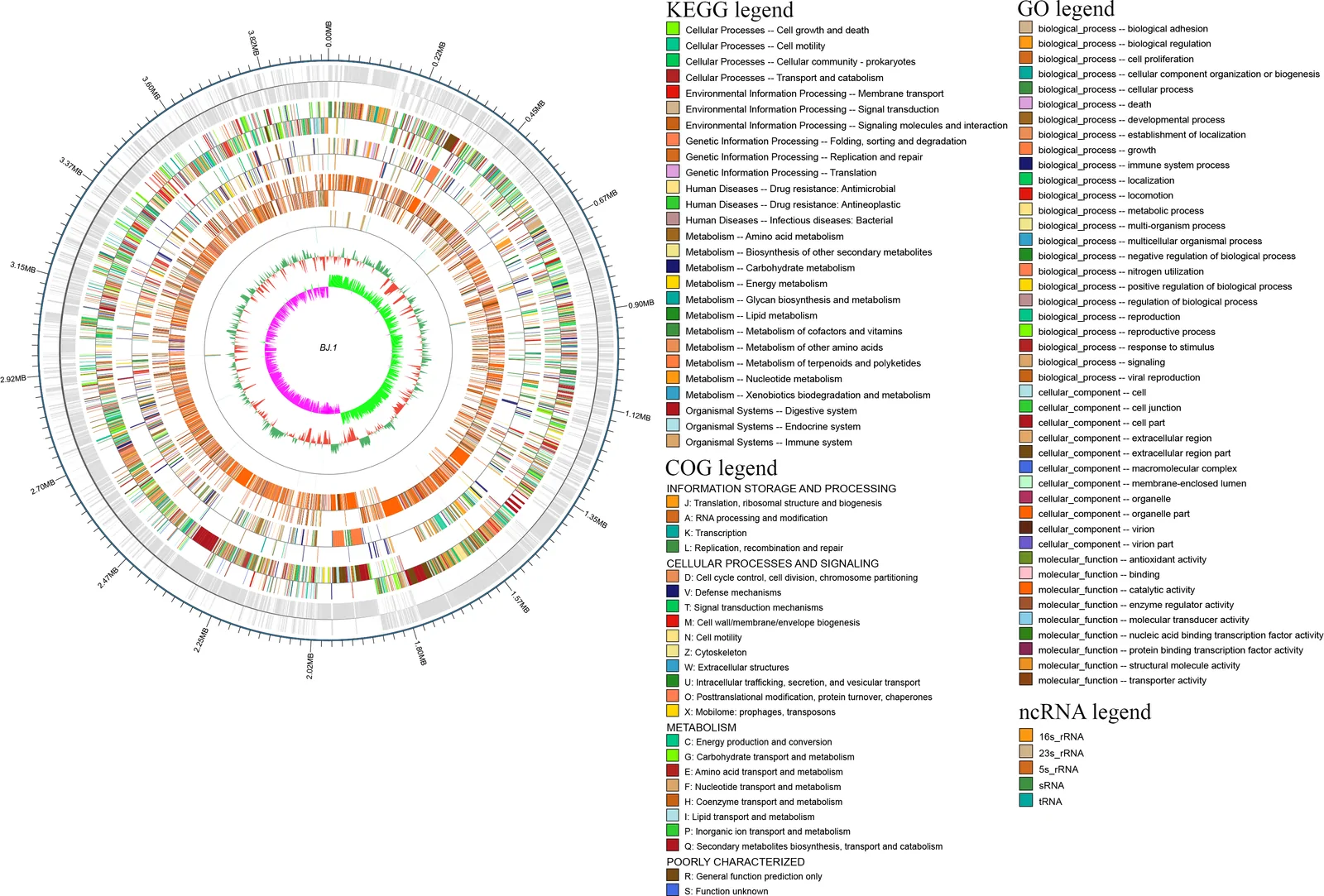
The circular genome map of *Bacillus velezensis* BJ1 consists of seven concentric circles representing, from the outside to the inside: (i) genome sequence, (ii) COG, (iii) KOG, (iv) eggNOG, (v) KEGG, (vi) GO, and (vii) ncRNA. The colors correspond to functional classification, and the legends indicate annotation categories.

Genome analysis revealed a complete circular chromosome in strain BJ1, with a total genome size of 3,974,433 bp and an average GC content of 47.28%. A total of 4,154 protein-coding genes were predicted, spanning 3,579,963 bp and accounting for approximately 90.07% of the genome. The intergenic and non-coding regions comprised 394,470 bp, with a GC content of 40.03%, representing 9.93% of the genome. A substantial proportion of protein-coding genes were functionally annotated across multiple databases, reflecting a well-characterized and functionally diverse proteome in the BJ1 strain (Fig. 2). Specifically, 3,310 genes were assigned high-confidence annotations in Swiss-Prot, while 3,961 genes were mapped to KEGG pathways (Fig. S1a), highlighting extensive metabolic and cellular capabilities. Additionally, 3,010 genes were classified into COG categories, indicating that genes in *B. velezensis* BJ1 were predominantly associated with metabolic and regulatory functions (Fig. S1c). The most abundant categories were amino acid transport and metabolism (302 genes), transcription (295 genes), carbohydrate transport and metabolism (248 genes), and translation, ribosomal structure, and biogenesis (224 genes), indicating strong metabolic versatility and active protein synthesis. Importantly, genes related to secondary metabolite biosynthesis, transport, and catabolism (86 genes) and defense mechanisms (97 genes) were well represented, supporting the genomic potential of BJ1 to produce antimicrobial compounds and compete with phytopathogens. Further, 2,651 genes were associated with Gene Ontology terms, indicating broad functional representation (Fig. S1b). The genome also encodes 511 transporter-related proteins (TCDB), suggesting strong nutrient acquisition and environmental adaptability. Notably, 344 genes were linked to host–pathogen interactions (PHI-base) and 187 to virulence-associated functions (VFDB), whereas relatively few genes were associated with antibiotic resistance (seven in ARDB and 51 in CARD). Furthermore, 87 secretory proteins, 84 type III secretion system-related genes, and 157 carbohydrate-active enzymes (CAZymes) were identified, supporting potential roles in host interaction, secretion, and carbohydrate metabolism. Overall, these annotations indicate that BJ1 possesses a functionally robust genome consistent with ecological fitness and potential biocontrol activity.

### Phylogenomic taxonomic identification of BJ1

**Fig 3:**
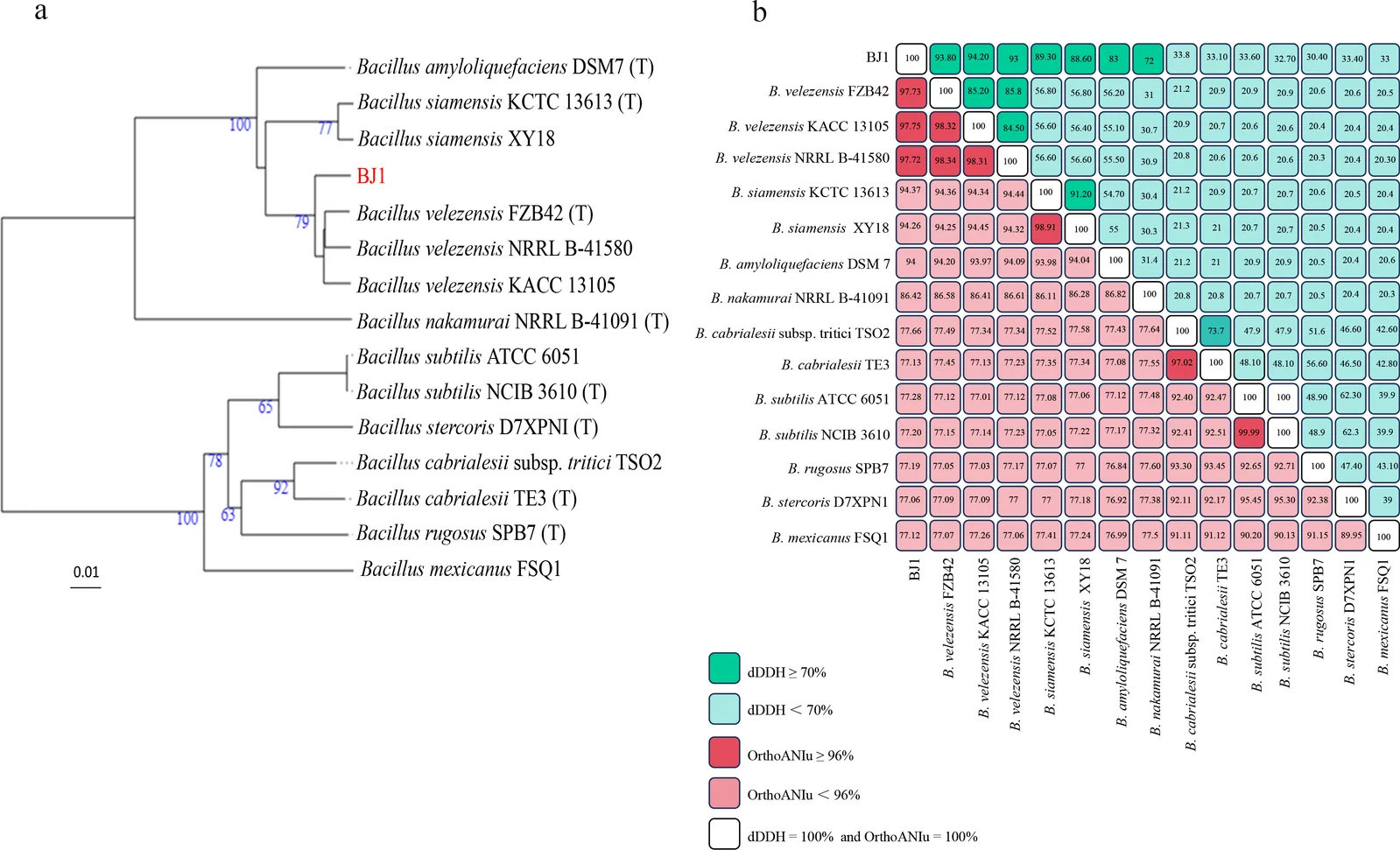
Taxonomic identification of *Bacillus velezensis* BJ1 strain. (**a**) Phylogenomic tree constructed using the type (strain) genome online server-TYGS, (**b**) pairwise OrthoANIu and dDDH values between the BJ1 genome and the selected type and reference strain genomes.

Whole-genome-based phylogenomic analysis performed using the TYGS platform assigned the strain BJ1 to the *Bacillus velezensis* lineage. As shown in the phylogenomic tree (Fig. 3a), BJ1 clustered closely with the *B. velezensis* type strain NRRL B-41580 (T), and reference strains KACC 13105 and FZB42, supported by high bootstrap values (≥75%). This taxonomic placement was further supported by digital DNA–DNA hybridization (dDDH) analysis (Fig. 3b), which revealed dDDH values of ≥93% between BJ1 and all examined *B. velezensis* reference strains, exceeding the established species delineation threshold of 70% (37, 43). Moreover, ANI analysis (Fig. 3b) showed that strain BJ1 exhibited ANI values greater than 97% with the *B. velezensis* type and reference strains NRRL B-41580 (T), KACC 13105, and FZB42, surpassing the widely accepted species cutoff of 95% (43) and thereby confirming its classification as *B. velezensis*. In contrast, ANI values calculated between BJ1 and other *Bacillus* species were below the species threshold, supporting clear genomic separation at the species level.

### *In silico* prediction of the biocontrol potential of BJ1

**Fig 4:**
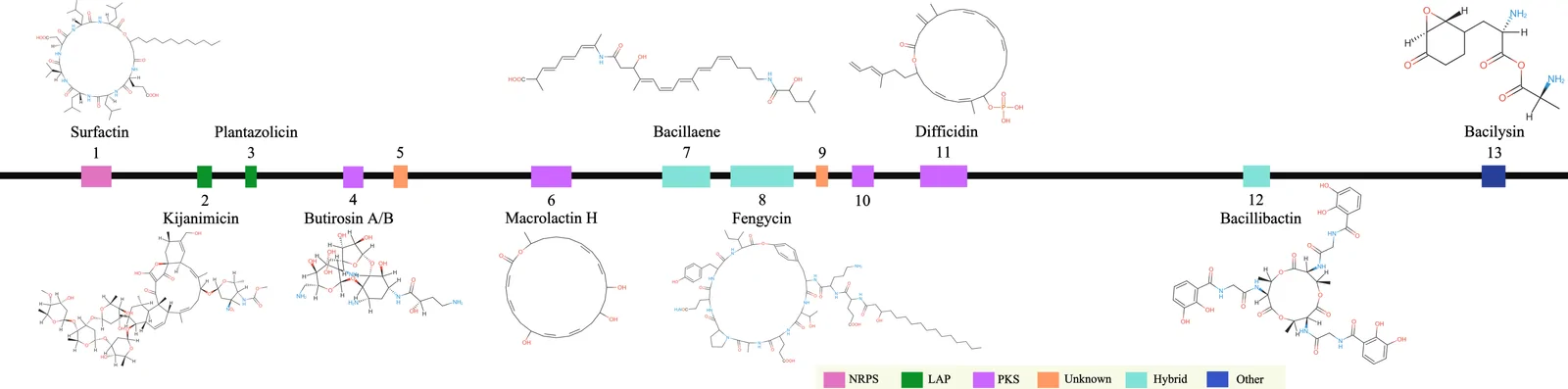
Genomic organization and distribution of *in silico* predicted secondary metabolite biosynthetic gene clusters in the BJ1 genome, with the corresponding predicted chemical structures of the antimicrobial compounds indicated in each cluster based on the MIBiG database.

Genome mining was performed using the antiSMASH platform to identify secondary metabolite biosynthetic gene clusters (BGCs) and characterize the secondary metabolite profile of *B. velezensis* BJ1, suggesting the genetic determinants that may contribute to its biocontrol properties (Table 1; Fig. 4; Fig. S2). The results suggested the presence of 13 biosynthetic gene clusters, corresponding to the production of 10 putative antimicrobial compounds with potential biocontrol activity, including surfactin, kijanimicin, plantazolicin, butirosin A/butirosin B, macrolactin H, bacillaene, fengycin, difficidin, bacillibactin, and bacilysin. The identified BGCs encompassed non-ribosomal peptide synthetase (NRPS) clusters, polyketide synthase (PKS) clusters, hybrid clusters, and other ribosomally synthesized and post-translationally modified peptide (RiPP) gene clusters. Among the predicted secondary metabolites, three compounds within PKS clusters (butirosin, macrolactin, and difficidin), two RiPP-type clusters (kijanimicin and plantazolicin), and surfactin within the NRPS cluster were identified (Table 1). Furthermore, the hybrid biosynthetic clusters were associated with bacillaene, fengycin, and bacillibactin. However, the terpene and T3PKS gene clusters identified in the BJ1 genome showed no detectable similarity to known reference clusters. Further, they could not be linked to any characterized secondary metabolites based on current database annotations. Most of the identified clusters showed high similarity to known reference clusters in the MIBiG database. However, kijanimicin and butirosin A/B showed low similarity to reference clusters (4% and 7%, respectively). In particular, the low-similarity clusters are predicted to be putatively novel biosynthetic gene clusters and have not been experimentally validated at the metabolite level.

**TABLE 1:** Biosynthetic gene clusters (BGCs) involved in the biosynthesis of secondary metabolites in the bacterial strain *Bacillus velezensis* BJ1 detected by antiSMASH server^*a*^

Cluster/region	Similarity	Predicted cluster position (from–to)		Synthetase type	Metabolite	Function
1	82%	304,714	370,121	NRPS	Surfactin	Antifungal activity, antibacterial activity, enhance colonization, induction of ISR
2	4%	584,139	613,252	Thiopeptide, LAP	Kijanimicin	Antibacterial activity
3	91%	696,357	719,534	RRE-containing, LAP	Plantazolicin	Antibacterial activity
4	7%	929,587	970,831	PKS-like	Butirosin A /butirosin B	Antibacterial activity
5	–^*b*^	1,052,866	1,073,606	terpene	–	–
6	100%	1,377,080	1,465,292	transAT-PKS	Macrolactin H	Antibacterial activity
7	100%	1,686,713	1,796,833	transAT-PKS, T3PKS, NRPS	Bacillaene	Antibacterial activity, induction of ISR
8	100%	1,854,296	1,992,125	NRPS, transAT-PKS, betalactone	Fengycin	Antifungal activity, induction of ISR
9	–	2,055,067	2,076,950	terpene	–	–
10	–	2,145,581	2,186,681	T3PKS	–	–
11	100%	2,302,170	2,408,336	transAT-PKS	Difficidin	Antibacterial activity
12	100%	3,074,489	3,126,280	NRP-metallophore, NRPS, RiPP-like	Bacillibactin	Antibacterial activity; nutrient uptake enhancement; siderophore production under iron-limited conditions; microbial competition.
13	100%	3,642,972	3,684,390	other	Bacilysin	Antibacterial activity

^*a*^ The predicted metabolite functions are based on antiSMASH annotations and published literature and were not experimentally validated in this study.

^*b*^ –, not applicable.

### *In vitro* antagonistic activity of BJ1 against phytopathogenic fungi

**Fig 5:**
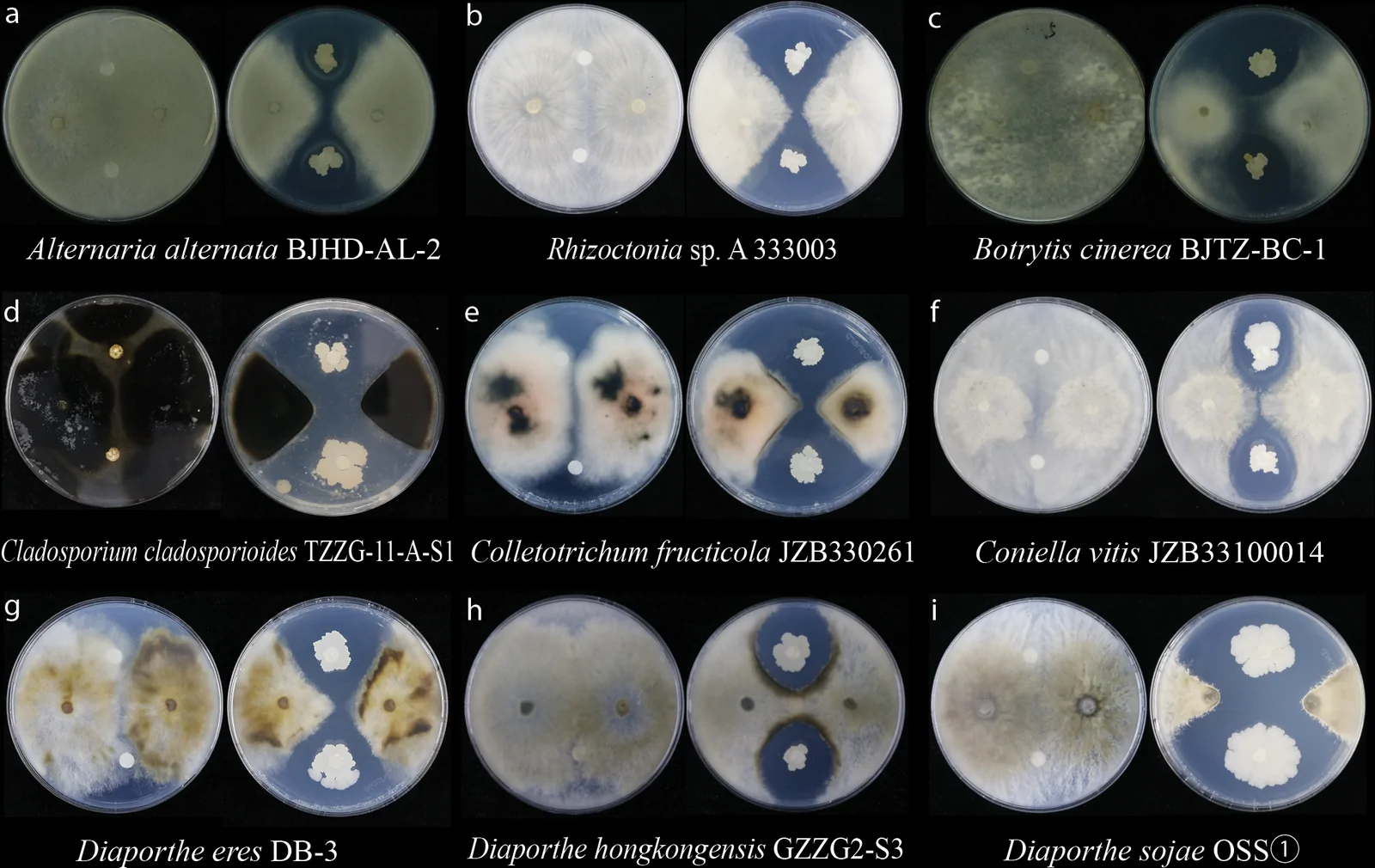
Representative dual culture assay plates showing the antagonistic activity of *Bacillus velezensis* BJ1 against selected phytopathogenic fungi. (a) *Alternaria alternata* BJHD-AL-2, (b) *Rhizoctonia* sp. A 333003, (c) *Botrytis cinerea* BJTZ-BC-1, (d) *Cladosporium cladosporioides* TZZG-11-A-S1, (e) *Colletotrichum fructicola* JZB330261, (f) *Coniella vitis* JZB33100014, (g) *Diaporthe eres* DB-3, (h) *Diaporthe hongkongensis* GZZG2-S3, and (i) *Diaporthe sojae* OSS①. In each pair, the left plate represents the control, while the right plate shows the antagonistic effect of strain BJ1.

The antagonistic activity of the BJ1 bacterial strain was evaluated against 59 isolates of fungal phytopathogens associated with eight fruit crops and vegetables using the dual culture assay under *in vitro* conditions (Table 2; Fig. 5; Table S1; Fig. S3). Clear inhibition zones were observed at the interaction interfaces between BJ1 and all tested fungal isolates. Overall, *B. velezensis* BJ1 exhibited broad-spectrum antifungal activity, with inhibition rates ranging from 53.76% to 98.45%. Based on the predefined susceptibility categories, six isolates showed very high susceptibility (>80% inhibition), 46 isolates showed high susceptibility (60–80% inhibition), seven isolates showed moderate susceptibility (40%–≤60% inhibition), and none of the tested isolates exhibited low susceptibility (<40% inhibition).

**TABLE 2:** Summary of the *in vitro* antagonistic activity of *Bacillus velezensis* BJ1 against fungal pathogenic genera in dual-culture assays^*a*^

Fungal genus	No. of isolates	Mean inhibition rate ± SE (%)	Inhibition range (%)	Susceptibility level
*Alternaria*	3	62.93 ± 4.19	58.59–71.31	Moderate–high
*Botryosphaeria*	6	65.38 ± 1.01	62.79–69.77	High
*Botrytis*	4	67.99 ± 2.09	64.95–73.91	High
*Cladosporium*	2	85.30 ± 0.08	85.22–85.37	Very high
*Colletotrichum*	12	69.22 ± 1.50	60.40–76.79	High
*Coniella*	2	71.32 ± 1.55	69.77–72.87	High
*Dactylonectria*	4	70.52 ± 2.23	66.67–74.70	High
*Diaporthe*	10	78.60 ± 2.91	69.77–98.45	High–very high
*Fusarium*	2	63.89 ± 1.85	62.04–65.74	High
*Lasiodiplodia*	3	62.01 ± 2.94	57.36–67.44	Moderate–high
*Neofusicoccum*	2	74.41 ± 2.32	72.09–76.74	High
*Neopestalotiopsis*	5	58.95 ± 2.15	53.76–64.29	Moderate–high
*Rhizoctonia*	3	71.06 ± 1.13	68.99–72.87	High
*Sclerotinia*	1	58.14	58.14	Moderate

^*a*^ Values are presented as mean inhibition percentage ± SE calculated from individual fungal isolates within each genus. The inhibition range indicates the minimum and maximum inhibition percentages among isolates of the same genus. Susceptibility levels were classified as low (<40%), moderate (40%–<60%), high (60%–≤80%), and very high (>80%). Detailed inhibition data for all 59 fungal isolates are provided in Table S1.

At the genus level, *Cladosporium* showed the highest susceptibility (85.30%), whereas *Neopestalotiopsis* (58.95%) and *Sclerotinia* (58.14%) exhibited the moderate mean inhibition rates (Table 2). The remaining genera showed mean inhibition rates in the moderate-to-high range. Variation in susceptibility among isolates within the same genus was observed, with *Diaporthe* showing the widest inhibition range (69.77%–98.45%), followed by *Colletotrichum* (60.40%–76.79%), *Neopestalotiopsis* (53.76–64.29%), and *Lasiodiplodia* (57.36–67.44%). In contrast, *Botryosphaeria* isolates exhibited relatively consistent susceptibility, with inhibition rates ranging from 62.79% to 69.77%.

At the species level, the highest inhibition was observed against *Diaporthe sojae* (98.45%), followed by *Cladosporium cladosporioides* (85.37%) and *D. eres* (80.62%), which were categorized as very highly susceptible (Table S1). Among grapevine-associated pathogens, BJ1 inhibited all tested isolates, with inhibition rates ranging from 57.36% to 98.45%; *D. sojae* showed the highest susceptibility, whereas *Lasiodiplodia pseudotheobromae* showed the lowest response. Further, cherry pathogens showed inhibition rates ranging from 58.59% to 89.15%, including five isolates with very high mean inhibition rates. Fungal pathogens associated with other crops, including strawberry, watermelon, eggplant, Chinese chives, and tomato, also exhibited moderate to high inhibition. Gray mold pathogen *Botrytis cinerea* from grapevine, tomato, and eggplant crops showed consistently high susceptibility (64.95%–73.91%).

### *In vivo* preventive and therapeutic efficacy of BJ1 against *Botrytis cinerea*

The potential biocontrol efficacy of the *B. velezensis* BJ1 bacterial isolate against the tomato gray mold pathogen (*B. cinerea*) was evaluated using a detached leaf inoculation assay by applying BJ1 fermentation at concentrations of 1 × 10^5^, 1 × 10^6^, 5 × 10^6^, and 1 × 10^7^ CFU/mL, with pre- and post-inoculation of *B. cinerea* mycelial discs, followed by measurement of lesion diameters on tomato leaves (Table 3; Fig. S4). Control efficiency generally increased with increasing BJ1 concentration in both preventive and therapeutic applications, while lesion diameters decreased correspondingly. The BJ1 treatment at 1 × 10⁷ CFU/mL was identified as the optimal concentration for controlling *B. cinerea*, as it consistently exhibited high biocontrol efficacy in both preventive (68.95%) and therapeutic (87.87%) applications. The control effect of BJ1 at 5 × 10⁶ CFU/mL in the therapeutic application (88.28%) was statistically comparable to that at 1 × 10⁷ CFU/mL. As the positive control, a commercial *Bacillus subtilis* wettable powder was used, which is a microbial fungicide registered for the control of fungal diseases affecting crop roots, leaves, and fruits. BJ1 treatments markedly reduced lesions caused by *B. cinerea*, showing comparable control efficiency to the commercial fungicide in the preventive application and significantly greater disease suppression in the therapeutic application, despite being applied at a lower concentration (Table 3). Overall, BJ1 treatments effectively suppressed the tomato gray mold disease compared with the sterile water-treated control, demonstrating strong biocontrol potential against *B. cinerea*. Furthermore, no phytotoxic effects were observed on tomato leaves treated with BJ1 suspensions at the tested concentrations in the absence of *B. cinerea* treatments.

**TABLE 3:** The preventive and therapeutic effects of *Bacillus velezensis* BJ1 against tomato gray mold disease caused by *Botrytis cinerea* in an *in vivo* leaf inoculation assay^*a*^

Treatment	Preventive effect			Therapeutic effect		
	Lesion diameter (av ± SE, cm)	Control efficiency (%)	Significance (α = 0.05)	Lesion diameter (av ± SE, cm)	Control efficiency (%)	Significance (α = 0.05)
1 × 10^7^ CFU/mL BJ1	0.43 ± 0.05	68.95	a	0.16 ± 0.02	87.87	a
5 × 10^6^ CFU/mL BJ1	0.65 ± 0.03	52.82	a	0.16 ± 0.01	88.28	a
1 × 10^6^ CFU/mL BJ1	0.68 ± 0.07	50.40	a	0.36 ± 0.01	73.22	b
1 × 10^5^ CFU/mL BJ1	1.11 ± 0.08	19.35	b	0.78 ± 0.03	41.42	c
1 × 10^8^ CFU/mL *Bacillus subtilis* WP (Positive control)	0.70 ± 0.08	49.19	a	1.06 ± 0.03	20.08	d
Sterile water (Negative control)	1.38 ± 0.11	–^*b*^	b	1.33 ± 0.02	–	e

^*a*^ Each treatment consisted of four biological replicates, with each replicate comprising six detached leaves placed in a separate crisper box. Data are expressed as mean ± SE. Different letters indicate significant differences based on Tukey’s HSD test (α = 0.05).

^*b*^ –, not applicable; control efficiency was calculated relative to the negative control treatment (sterile water).

### *In planta* therapeutic efficacy of BJ1 against *Botrytis cinerea*

To further validate the disease-controlling capability of the BJ1 bacterial strain against *B. cinerea*, an *in planta* assay was conducted under greenhouse conditions (Table 4). Three different concentrations of BJ1 (5 × 10^7^, 1 × 10^8^, 2 × 10^8^ CFU/mL) were applied as therapeutic treatments for *B. cinerea*-infected tomato plants for subsequent evaluation of disease control efficacy. All BJ1 treatments significantly reduced the incidence of gray mold disease in tomato plants compared with the negative control (*P* < 0.01), achieving control efficacies ranging from 72.38% to 80.48%, which were higher than that of the positive control. Notably, BJ1 applied at 2 × 10⁸ CFU/mL resulted in the lowest disease incidence (1.23%) and the highest control efficacy (80.48%). Although the positive control (*B. subtilis*) also significantly reduced the disease incidence compared with the negative control (sterile water), its efficacy (66.48%) was lower than that of BJ1 treatments. These results demonstrate that the BJ1 strain has strong potential as a biocontrol agent for managing tomato gray mold disease.

**TABLE 4:** Disease control effect of *Bacillus velezensis* BJ1 against *Botrytis cinerea* on tomato plants under greenhouse conditions^*a*^

Treatment	Average rate of diseased plants ± SE (%)	Average inhibition rate ± SE (%)	Significance	
			0.05	0.01
5 × 10^7^ CFU/mL BJ1	1.74 ± 0.17	72.38 ± 1.59	b	B
1 × 10^8^ CFU/mL BJ1	1.50 ± 0.08	76.05 ± 0.75	b	AB
2 × 10^8^ CFU/mL BJ1	1.23 ± 0.11	80.48 ± 0.52	a	A
1.56 × 10^8^ CFU/mL *Bacillus subtilis* WP (Positive control)	2.09 ± 0.04	66.48 ± 1.85	c	C
Sterile water (Negative control)	6.32 ± 0.52	–^*b*^	d	D

^*a*^ Values are the mean of four independent biological replicates (*n* = 4). Means followed by different letters are significantly different according to Tukey’s HSD test (lowercase: α = 0.05; uppercase: α = 0.01).

^*b*^ –, not applicable; average inhibition rate was calculated relative to the negative control treatment (sterile water).

## DISCUSSION

Biological control using naturally occurring microorganisms represents a promising and sustainable approach to plant disease management in agriculture. In this study, we employed an integrated approach combining *in silico* genome mining, laboratory, and greenhouse experiments to explore the biocontrol potential of *B. velezensis* BJ1. Phylogenomic analysis, together with ANI and dDDH values, confirmed the taxonomic identity of strain BJ1. Compared with traditional methods, whole-genome sequencing provides a more accurate framework for bacterial taxonomy, enabling high-resolution species delineation while offering deeper insights into the genetic, physiological, and functional potential of a strain (44).

Genome mining suggested that *B. velezensis* BJ1 possesses a diverse repertoire of biosynthetic gene clusters (BGCs), indicating a remarkable genetic potential for secondary metabolite production. In total, 13 biosynthetic gene clusters (BGCs) were identified, including non-ribosomal peptide synthetase (NRPS), polyketide synthase (PKS), hybrid NRPS–PKS, and ribosomally-synthesized and post-translationally modified peptide (RiPP) clusters. NRPS and PKS systems function as large multienzyme complexes that assemble diverse bioactive compounds from amino acid and malonyl-derived precursors, many of which are known to inhibit phytopathogenic fungi and bacteria (15, 20, 45–47). Among the identified clusters, several corresponded to the biosynthesis of well-characterized antimicrobial compounds, including surfactin, fengycin, bacillaene, difficidin, macrolactin H, bacilysin, bacillibactin, and plantazolicin. These metabolites are widely implicated in antibiosis and exhibit broad-spectrum antimicrobial activity (48–53). Apart from these well-characterized clusters, the BJ1 strain harbored BGCs showing low similarity to known MIBiG entries, including kijanimicin-like (4% similarity) and butirosin A/B-like (7% similarity) clusters. These clusters likely represent highly divergent or potentially novel biosynthetic pathways. In addition, terpene and T3PKS biosynthetic gene clusters identified in the BJ1 genome showed no detectable similarity to characterized MIBiG reference clusters, suggesting the presence of uncharacterized biosynthetic pathways that may be underrepresented in current databases. Therefore, future experimental validation is required to confirm their metabolite identity and biological activity. Among the identified metabolites, fengycin and surfactin, both cyclic lipopeptides, are particularly important for antifungal activity, as they disrupt pathogen cell membranes through pore formation, leading to osmotic imbalance and cell death (52–54). Moreover, surfactin functions as a biosurfactant that facilitates bacterial motility, biofilm development, and surface colonization (54, 55). Bacillaene is an effective antimicrobial compound with broad-spectrum antibacterial and antifungal activities, contributing to microbial self-survival, competitive interactions with other microorganisms, and biofilm formation (56). Macrolactins exhibit a wide range of biological activities, including antibacterial, antifungal, antiviral, anticancer, anti-inflammatory, and anti-angiogenic effects, and macrolactins produced by *B. velezensis* have been shown to directly inhibit pathogen cell division and downregulate their virulence-associated genes (49). Bacilysin exhibits broad inhibitory activity against fungal and bacterial pathogens as well as algae, mediated by its anticapsin moiety, which inhibits glucosamine-6-phosphate synthase, thereby blocking cell wall synthesis and leading to microbial cell lysis (57, 58). Transcriptomic analyses of previous studies further indicated that bacilysin modulates genes involved in metabolic adaptation, antimicrobial biosynthesis, metal (zinc and iron) homeostasis, sporulation and germination, motility and biofilm formation, biofilm-associated toxin production, and stress responses (48). In addition, bacillibactin functions as a siderophore, enhancing iron acquisition under iron-limited conditions and suppressing competitive microorganisms either by iron sequestration or direct growth inhibition (50, 51). Difficidin has been predominantly reported in *B. velezensis* strains and is known to exert bactericidal activity via inhibition of protein synthesis (59, 60). Plantazolicin acts as an antibacterial compound that disrupts the bacterial cell envelope by depolarizing the membrane and preferentially accumulating at vulnerable membrane regions, leading to cell lysis (61). This selective activity is associated with membrane stress responses and interactions with membrane lipids such as cardiolipin (61). Overall, the presence of multiple antimicrobial biosynthetic gene clusters in BJ1 provides a genetic basis for its antimicrobial activity and supports its potential role in biocontrol applications.

*B. velezensis* BJ1 bacterial strain demonstrated its broad-spectrum antagonistic activity, exhibiting a significant growth inhibition of 59 different fungal phytopathogen isolates in the dual culture assay (Table 2; Table S1; Fig. 5; Fig. S3). Similarly, different *B. velezensis* strains have shown strain-specific biocontrol activity against diverse fungal pathogens (62). In our study, the *B. velezensis* BJ1 strain showed the strongest antagonistic activity against the *Diaporthe sojae* OSS① strain (98.45%) from the *Vitis vinifera* L. (grapevine) host plant, which is higher than the inhibition rate reported in previous studies (12). Additionally, *D. sojae* B2 JZB320190, *Cladosporium cladosporioides* TZZG-11-A-S1, *C. cladosporioides* TZZG-6-A-S1, *D. sojae* JZB320189, and *D. eres* TZZG36-S1 isolates exhibited growth inhibition rates exceeding 80%. The use of biocontrol agents is important to manage the diseases caused by *Diaporthe* and *Cladosporium* species, as these widespread phytopathogens are responsible for significant crop losses worldwide, especially in viticulture (63, 64). All tested pathogens exhibited growth inhibition rates greater than 53%, highlighting the broad-spectrum pathogen inhibition ability of BJ1, which can be attributed to a combination of mechanisms, including the rapid growth trait with competition for essential resources such as nutrients, space, moisture, and secretion of lytic enzymes and antimicrobial compounds targeting a wide range of modes of action (9, 65, 66). Pathogenic mycelial growth inhibition before physical contact suggests that the antifungal activity of BJ1 is primarily mediated by diffusible antibiotic compounds (67, 68). Once attached to the pathogen hyphae, microbial antagonists can further restrict fungal growth through more efficient nutrient uptake and protect against infection via the production of antifungal compounds (69, 70). Although the strain may carry genes for antimicrobial production, actual expression can be influenced by factors such as nutrient composition and other culture conditions (71, 72). Therefore, a combination of assays is essential to reliably assess biocontrol potential, as outcomes can vary with strain differences, application methods, concentrations, and assay conditions (9).

To further validate the biocontrol ability of *B. velezensis* BJ1, *Botrytis cinerea* was used as the target pathogen. *B. cinerea* is a highly virulent necrotrophic pathogen that causes gray mold disease in over 1,400 plant species, and results in significant pre- and post-harvest losses (73). In the dual culture assays, *B. cinerea* strains isolated from tomato, grapes, and eggplant exhibited high growth inhibition rates of 67.97%, 64.95%, and 65.12%, respectively, when treated with *B. velezensis* BJ1. These results stand in contrast to previous research, where various other *B. velezensis* strains reported a range of growth inhibition rates of *B. cinerea*, such as 20%-40% (74), 50% (30), 47% (75), 58% (76), 60% (46), 72.23% (77), and 75.22% (78) under *in vitro* conditions, indicating strain-specific inhibition rates. Previous transcriptomic analyses have shown that exposure to *B. velezensis* causes extensive changes in *B. cinerea* gene expression, particularly in pathways related to carbon metabolism (starch and sucrose, galactose, fatty acid, fructose, and mannose metabolism pathways), energy production (cellular ATP synthesis pathways), cell wall biosynthesis, membrane integrity, and antioxidant defense (59). In addition to direct inhibition, cell-free culture filtrates of *B. velezensis* strains have been shown to inhibit *B. cinerea* by up to 76.9%, indicating that secreted metabolites in culture filtrate play a key role in suppressing mycelial growth, spore germination, and altering fungal morphology (79). Different biocontrol assays evaluate various modes of action of the biocontrol agent. The antagonistic dual culture assay with live organisms evaluates direct interactions between the pathogen and biocontrol strain, indicating inhibition by bioactive compounds and resource competition (9). Additionally, the cell-free antagonistic supernatant method emphasizes the effects of secreted metabolites, such as enzymes, lipopeptides, and polyketides (80).

Our study provides insights into the effectiveness of *B. velezensis* BJ1 in controlling tomato gray mold disease, examining both preventive and therapeutic approaches. The preventive treatment involved applying BJ1 before fungal inoculation, analogous to plant protection measures aimed at disease prevention, while the therapeutic treatment was applied after pathogen infection, resembling a curative disease management strategy. Therapeutic control provides prompt, targeted intervention after symptom onset, helping to mitigate disease impact and reduce the risk of pathogen resistance, although it may not completely prevent crop damage (9). In contrast, preventive methods reduce disease incidence, limit pathogen spread along the agricultural value chain, and allow biocontrol agents to establish early, preempt resources, and occupy ecological niches before pathogen colonization, thereby enhancing overall efficacy, though they require upfront investment and continuous monitoring (9, 81). Consistent with our findings, previous studies have reported that various *Bacillus* strains, including *B. velezensis*, effectively suppressed *B. cinerea* in tomato plants when applied preventively (41, 82–84). Similarly, pre-treatment with *B. velezensis* L33a in detached tomato fruit assays significantly reduced gray mold symptoms by suppressing pathogenicity genes of *B. cinerea* and inducing salicylic acid (SA), jasmonic acid (JA), and antioxidant defense pathways in hosts, highlighting the multifaceted biocontrol mechanisms of *B. velezensis* strains (78).

Whole-plant assays under greenhouse conditions demonstrate the ability of a biocontrol agent to suppress disease occurrence and development under realistic environmental conditions by reflecting the tripartite interaction among the biocontrol candidate, the phytopathogen, and the plant host, while also indicating its capacity to persist on plant tissues and exert disease suppression through multiple biocontrol mechanisms beyond those observed in *in vitro* assays (9, 85). In the present study, we observed significant disease control in treated plants compared with the negative control, together with a clear dose-dependent disease suppression in both detached-leaf inoculation and *in planta* assays, where higher concentrations were consistently more effective. This enhanced efficacy at higher cell concentrations may be attributed to increased competition for space and nutrients, as well as stronger antagonistic interactions that suppress pathogen growth (75, 86). Among the tested treatments, BJ1 applied at 2 × 10^8^ CFU/mL resulted in the lowest disease incidence (1.23%) and the highest disease inhibition rate (80.48%). This disease suppression level was greater than that reported for several previously described *B. velezensis* strains, including NKG-1 (60%), 5YN8 (58.44%), and DSN012 (56.26%), under greenhouse conditions (15).

Previous studies have often reported lower biocontrol efficacy in *in planta* experiments compared with *in vitro* assays (87–89), likely due to environmental factors, inoculation methods, colonization efficiency, and differences in antifungal compound production (9, 90). Conversely, some biocontrol strains exhibit strong disease suppression in *in planta* experiments despite showing little or no inhibitory activity in *in vitro* assays (91). In the present study, the greater suppression of *B. cinerea* observed *in planta* may be attributed to the rapid colonization and establishment of *B. velezensis* BJ1 on tomato plant tissues, together with the activation of antagonistic traits and host-mediated systemic resistance (92). Further, genome mining suggested surfactin- and bacillaene-like secondary metabolite gene clusters in BJ1, which are known to support motility, biofilm formation, and surface colonization, thereby potentially enhancing persistence on the host and overall biocontrol efficacy (54, 55, 80). In addition to these intrinsic traits, the method of biocontrol agent application also plays an important role in disease management by influencing the distribution, persistence, and establishment of biocontrol agents on plant surfaces, thereby affecting both direct antagonism and induction of plant systemic resistance (93–95). In this study, BJ1 was applied as a foliar spray, which provides direct and uniform coverage of above-ground tissues, facilitating immediate antagonistic interactions and enhancing local biocontrol activity (96). Consistent with our findings, foliar applications of *B. velezensis* strains in previous studies have also effectively suppressed gray mold disease in different hosts, including strain 160 against gray mold of pomegranate (97) and strain SL-6 against postharvest gray mold of pears (98).

Overall, the present study provides important insights into the biocontrol potential of *B. velezensis* BJ1 through integrated *in vitro*, *in vivo*, *in planta*, and genome mining analyses. While these findings highlight the functional and genomic basis of its biocontrol activity, further work is required to fully elucidate the underlying mechanisms. In particular, validation of predicted secondary metabolites through targeted metabolite profiling and purification, coupled with bioassay-guided fractionation, would help confirm the specific compounds responsible for antifungal activity. In addition, systems-level approaches such as transcriptomic and metabolomic analyses may provide deeper insights into regulatory networks and metabolic responses associated with antimicrobial activity and plant–microbe interactions. From an application standpoint, optimization of fermentation and formulation stability, together with field validation under diverse environmental conditions, would be important to assess the consistency of performance and commercial applicability of BJ1-based biofungicides. Moreover, evaluation of compatibility with existing integrated pest management practices and assessment of biosafety, including effects on non-target organisms and environmental persistence, would support future development for practical application.

In conclusion, *B. velezensis* BJ1 exhibited strong broad-spectrum antifungal activity against multiple phytopathogens, including *Botrytis cinerea*, with effective performance in both preventive and curative applications for tomato gray mold disease control. Genomic analyses further suggested the genetic capacity of the BJ1 strain to produce diverse antimicrobial compounds, supporting the high biocontrol efficacy (>80%) observed at the plant level. Collectively, these findings demonstrated *B. velezensis* BJ1 as a promising and environmentally friendly candidate for development as a sustainable biocontrol agent for plant disease management.

## Data Availability

The whole-genome sequence of *Bacillus velezensis* BJ1 has been deposited in the Genome Warehouse of the National Genomics Data Center, Beijing Institute of Genomics, Chinese Academy of Sciences/China National Center for Bioinformation, under accession number GWHERFU00000000 and is publicly available at https://ngdc.cncb.ac.cn/gwh. The *B. velezensis* BJ1 strain is preserved in the China General Microbiological Culture Collection Center (CGMCC), Institute of Microbiology, Chinese Academy of Sciences, under accession number CGMCC 24113.

## SUPPLEMENTAL MATERIAL

Supplementary data spectrum.00944-26-s0001.tiff Functional genome annotation of *Bacillus velezensis* BJ1 based on KEGG pathway annotation.

Supplementary data spectrum.00944-26-s0002.tiff Functional genome annotation of *Bacillus velezensis* BJ1 based on Gene Ontology (GO) classification.

Supplementary data spectrum.00944-26-s0003.tiff Functional genome annotation of *Bacillus velezensis* BJ1 based on COG functional classification.

Fig. S2 spectrum.00944-26-s0004.tiff Comparison of predicted BGCs from *B. velezensis* BJ1 with similar known gene clusters deposited in the MIBiG database using antiSMASH.

Fig. S3 spectrum.00944-26-s0005.tif Antagonistic activity of *Bacillus velezensis* BJ1 against phytopathogenic fungi in the dual culture assay. In each pair, the left plate indicates the control, and the right plate shows the antagonistic activity of strain BJ1.

Fig. S4 spectrum.00944-26-s0006.tiff Therapeutic and protective effects of *Bacillus velezensis* BJ1 against gray mold disease caused by *Botrytis cinerea* on tomato leaves 48 h after treatment.

Supplementary data spectrum.00944-26-s0007.docx *In vitro* antagonistic activity of *Bacillus velezensis* BJ1 against 59 isolates of plant-pathogenic fungi, expressed as inhibition rates (%) of mycelial growth in a dual-culture assay.

ASM does not own the copyrights to Supplemental Material that may be linked to, or accessed through, an article. The authors have granted ASM a non-exclusive, world-wide license to publish the Supplemental Material files. Please contact the corresponding author directly for reuse.
