# Supplementary material for: Integrated assays with in silico analyses indicated the biocontrol potential of Bacillus velezensis BJ1 against fungal phytopathogens
Source: Microbiol Spectr. 2026 Oct 14;14(11):e00944-26. doi: 10.1128/spectrum.00944-26 (PMC13729146; doi:10.1128/spectrum.00944-26)
Supplement: Supplementary file 1 [file spectrum.00944-26-s0007.docx]

**Supplemental material:**

**Table S1.** *In vitro* antagonistic activity of *Bacillus velezensis* BJ1 against 59 isolates of plant-pathogenic fungi, expressed as inhibition rates (%) of mycelial growth in a dual-culture assay.

| Host | Diseases | Pathogens | Inhibition rate ± SE (%) | Susceptibility level |
| --- | --- | --- | --- | --- |
| Grapevine | Anthracnose | *Colletotrichum viniferum* YT | 65.56±1.57 | High |
|  |  | *Colletotrichum viniferum* BT-2 | 69.45±1.31 | High |
|  | Black foot disease | *Dactylonectria alcacerensis* JZB3310014 | 74.03±3.67 | High |
|  |  | *Dactylonectria alcacerensis* JZB3310013 | 74.70±2.95 | High |
|  |  | *Dactylonectria macrodidyma* JZB3310008 | 66.67±2.77 | High |
|  |  | *Dactylonectria torresensis* JZB3310015 | 66.67±3.89 | High |
|  | Botryosphaeria Dieback | *Botryosphaeria dothidea* GXZYD2s | 62.79±1.90 | High |
|  |  | *Botryosphaeria dothidea* GS-01S② | 65.89±2.90 | High |
|  |  | *Lasiodiplodia pseudotheobromae* WHSB4P03S | 57.36±1.10 | Moderate |
|  |  | *Lasiodiplodia theobromae* CSS-01S | 67.44±1.90 | High |
|  |  | *Lasiodiplodia theobromae* GX-5-5 | 61.24±3.95 | High |
|  |  | *Neofusicoccum parvum* SCCDJF-01S | 76.74±1.90 | High |
|  |  | *Neofusicoccum parvum* AH-3-1-01S | 72.09±1.90 | High |
|  | Diaporthe dieback | *Diaporthe eres* HS3-1 | 69.81±1.33 | High |
|  |  | *Diaporthe eres* DB-3 | 75.73±1.37 | High |
|  |  | *Diaporthe sojae* OSS① | 98.45±1.10 | Very high |
|  |  | *Diaporthe sojae* GG-6 | 77.31±2.06 | High |
|  | Gray mold | *Botrytis cinerea* SX | 64.95±1.46 | High |
|  |  | *Botrytis cinerea* HB-2 | 73.91±3.55 | High |
|  | Shoot Rot | *Neopestalotiopsis* sp. JL-4-⑤ | 64.29±1.26 | High |
|  |  | *Neopestalotiopsis* sp. GXXA2-1 | 58.06±2.63 | Moderate |
|  |  | *Neopestalotiopsis* sp. PTP038 | 63.57±1.10 | High |
|  | White Rot | *Coniella vitis* clmsl33-2F | 69.77±1.90 | High |
|  |  | *Coniella vitis* JZB33100014 | 72.87±1.10 | High |
| Cherry | Gummosis Disease | *Botryosphaeria dothidea* JZB310198 | 64.34±1.10 | High |
|  |  | *Botryosphaeria dothidea* JZB310202 | 63.57±1.10 | High |
|  |  | *Botryosphaeria dothidea* GZZG7-A-S1 | 65.89±2.19 | High |
|  |  | *Botryosphaeria dothidea* SYZG-13-A-S1 | 69.77±1.90 | High |
|  | Leaf Spot | *Alternaria alternata* A1 | 58.88±2.64 | Moderate |
|  |  | *Alternaria alternata* A2 | 58.59±5.15 | Moderate |
|  |  | *Colletotrichum aenigma* JZB330210 | 71.20±1.96 | High |
|  |  | *Colletotrichum aenigma* JZB330211 | 76.79±5.50 | High |
|  |  | *Colletotrichum fructicola* JZB330259 | 61.25±1.77 | High |
|  |  | *Colletotrichum fructicola* JZB330261 | 74.40±2.26 | High |
|  |  | *Colletotrichum gloeosporioides* JZB330237 | 67.86±2.19 | High |
|  |  | *Colletotrichum gloeosporioides* JZB330239 | 70.00±2.23 | High |
|  |  | *Colletotrichum sojae* JZB330291 | 68.85±5.05 | High |
|  |  | *Colletotrichum sojae* JZB330293 | 76.38±3.86 | High |
|  |  | *Cladosporium cladosporioides* TZZG-6-A-S1 | 85.22±1.23 | Very high |
|  |  | *Cladosporium cladosporioides* TZZG-11-A-S1 | 85.37±1.99 | Very high |
|  |  | *Diaporthe eres* TZZG36-S1 | 80.62±3.95 | Very high |
|  |  | *Diaporthe eres* SDZG35-C-S2 | 74.02±1.93 | High |
|  |  | *Diaporthe hongkongensis* GZZG2-S1 | 70.54±1.10 | High |
|  |  | *Diaporthe hongkongensis* GZZG2-S3 | 69.77±1.90 | High |
|  |  | *Diaporthe sojae* JZB320189 | 80.62±2.90 | Very high |
|  |  | *Diaporthe sojae* B2 JZB320190 | 89.15±1.10 | Very high |
| Strawberry | Anthracnose | *Colletotrichum siamense* 330161 | 68.47±2.55 | High |
|  | Root Rot | *Rhizoctonia* sp. A 333003 | 71.32±1.10 | High |
|  |  | *Rhizoctonia* sp. A 333002 | 72.87±1.10 | High |
|  |  | *Rhizoctonia* sp. G 333001 | 68.99±1.10 | High |
|  |  | *Fusarium oxysporum* LGS15 | 62.04±1.31 | High |
|  |  | *Neopestalotiopsis rosae* 340066 | 53.76±1.52 | Moderate |
|  |  | *Neopestalotiopsis rosae* 340065 | 55.06±3.18 | Moderate |
| Watermelon | Fusarium Wilt | *Fusarium oxysporum* Xgld | 65.74±3.46 | High |
| Chili | Anthracnose | *Colletotrichum acutatum* BJYH-CG-3 | 60.40±1.40 | High |
| Chinese chives | Sclerotinia Stem Rot | *Sclerotinia sclerotiorum* BJTZ-SS-1 | 58.14±1.90 | Moderate |
| Eggplant | Gray Mold | *Botrytis cinerea* BJTZ-BC-1 | 65.12±1.90 | High |
| Tomato | Early Blight | *Alternaria alternata* BJHD-AL-2 | 71.31±1.16 | High |
|  | Gray Mold | *Botrytis cinerea* BJFS-BC-36 | 67.97±1.10 | High |

Notes: Values are mean ± standard error (SE) of three independent biological replicates (n=3). Inhibition levels were classified as low (<40%), moderate (40–<60%), high (60–≤80%), and very high (>80%).
